# Ecotoxicological Evaluation of Safener and Antimicrobial Additives in Isoxaflutole-Based Herbicide Formulations

**DOI:** 10.3390/toxics12040238

**Published:** 2024-03-24

**Authors:** Eszter Takács, Diána Lázár, Augustine Siakwa, Szandra Klátyik, Mária Mörtl, László Kocsányi, Attila Barócsi, Sándor Lenk, Edina Lengyel, András Székács

**Affiliations:** 1 Agro-Environmental Research Centre, Institute of Environmental Sciences, Hungarian University of Agriculture and Life Sciences, Páter Károly u. 1., H-2100 Gödöllő, Hungary; siakwaa@yahoo.com (A.S.); klatyik.szandra@uni-mate.hu (S.K.); mortl.maria@uni-mate.hu (M.M.); szekacs.andras@uni-mate.hu (A.S.); 2 Limnology Research Group, Center of Natural Science, University of Pannonia, Egyetem u. 10., H-8200 Veszprém, Hungary; lazar.diana@blki.hun-ren.hu (D.L.); lengyel.edina@mk.uni-pannon.hu (E.L.); 3 Aquatic Botany and Microbial Ecology Research Group, HUN-REN-BLKI, Klebelsberg Kuno u. 3, H-8237 Tihany, Hungary; 4 Department of Atomic Physics, Institute of Physics, Budapest University of Technology and Economics, Műegyetem rkp. 3., H-1111 Budapest, Hungary; kocsanyi.laszlo@ttk.bme.hu (L.K.); barocsi.attila@ttk.bme.hu (A.B.); lenk.sandor@ttk.bme.hu (S.L.); 5 Limnoecology Research Group, ELKH-PE, Egyetem u. 10, H-8200 Veszprém, Hungary

**Keywords:** isoxaflutole, Merlin Flexx, Merlin WG75, cyprosulfamide, 1,2-benzisothiazol-3(2H)-one, safener, combined effect, herbicide, ecotoxicology, fluorescence

## Abstract

The environmental load by isoxaflutole and its formulated herbicide products has increasingly become apparent because, after the ban of atrazine, isoxaflutole has become its replacement active ingredient (a.i.). Obtaining information regarding the fate of this a.i. in environmental matrices and its ecotoxicological effects on aquatic organisms is essential for the risk assessment of the herbicide. In this study, the effects of Merlin Flexx- and Merlin WG75 formulated isoxaflutole-based herbicide products and two selected additives (cyprosulfamide safener and 1,2-benzisothiazol-3(2H)-one antimicrobial agent) were investigated on *Raphidocelis subcapitata* in growth inhibition assays. In ecotoxicological tests, two conventional (optical density and chlorophyll-a content) and two induced fluorescence-based (Fv*/Fp: efficiency of the photosystem PSII and Rfd* changes in the observed ratio of fluorescence decrease) endpoints were determined by UV-spectrophotometer and by our FluoroMeter Module, respectively. Furthermore, dissipation of isoxaflutole alone and in its formulated products was examined by an HPLC-UV method. In ecotoxicological assays, the fluorescence-based Rfd* was observed as the most sensitive endpoint. In this study, the effects of the safener cyprosulfamide and the antimicrobial agent 1,2-benzisothiazol-3(2H)-one on *R. subcapitata* is firstly reported. The results indicated that the isoxaflutole-equivalent toxicity of the mixture of the isoxaflutole–safener–antimicrobial agent triggered lower toxicity (EC_50_ = 2.81 ± 0.22 mg/L) compared to the individual effect of the a.i. (EC_50_ = 0.02 ± 0.00 mg/L). The Merlin Flexx formulation (EC_50_ = 27.04 ± 1.41 mg/L) was found to be approximately 50-fold less toxic than Merlin WG75, which can be explained by the different chemical characteristics and quantity of additives in them. The additives influenced the dissipation of the a.i. in Z8 medium, as the DT_50_ value decreased by approximately 1.2- and 3.5-fold under light and dark conditions, respectively.

## 1. Introduction

Agricultural micropollutants (pesticide active ingredients, co-formulants, and other additives, mycotoxins, and fertilizers) can leach out from soil and contaminate surface water and drinking water supplies, and thus exert direct and indirect adverse effects on aquatic organisms and human health [1,2,3,4,5]. Agriculture, expanding in an effort to provide food for the growing and increasingly consuming human population, is forecasted to dramatically contribute to biodiversity collapse and become a major driver of global environmental change by 2050 [6,7,8,9]. Deterioration of water quality is also a key problem related to these contaminants [1,3,10,11], and consequently, internationally harmonized water quality indicators are in use to describe the state of water quality in agricultural areas and define the contribution of nutrient and pesticide pollution originating from agricultural activities [12].

Pesticide products exert their targeted activity by their active ingredients (a.i.s) interfering with key physiological processes in the pest organisms. In addition to the a.i.s, these products also contain various additives that can facilitate the stability, distribution, or efficacy of the a.i. or modify its adhesivity or other physicochemical characteristics. Three groups of additives need to be mentioned for the purpose of this study. Surfactants reduce the surface tension of the aqueous solution of the a.i., thus facilitating the spreading of the formulation used in plant protection as a film layer on the surface, thus aiding the absorption of the a.i. [13]. Antidotes (safeners) reduce or eliminate the phytotoxic effect of the a.i. on certain plants mostly by enhancing metabolic enzymes in these plants that can rapidly degrade the a.i. of pesticide formulations. The use of antidotes has several advantages in agricultural weed control: (i) selective eradication of weeds in botanically related crops, (ii) the use of non-selective herbicides for selective weed control, and (iii) the use of persistent soil herbicides by enhancing their degradation. An important drawback, however, is that the antidote may facilitate the emergence of weed resistance, as increased herbicide metabolism is a key mechanism in the development of not target-site-based weed resistance [14]. Antimicrobial and/or antifungal additives (preservatives) protect the a.i. from microbial degradation or unexpected chemical transformation on the plant surface, thus promoting enhanced efficacy of the formulation [15]. As seen, antidotes (safeners) and antimicrobial additives are biologically active components. Therefore, they are not typical additives, as additives are, in principle, inert regarding the pesticidal mode of action of the a.i., and therefore, formulation substances used to be evaluated as inert components in the regulatory risk assessment process. Certain components in pesticide formulations applied to animals, including the sodium salts of sulfomethylated lignosulfonic acid suspension/emulsion stabilizers, are to date exempt from the requirement of tolerance in the U.S. as “inert ingredients” [16]. Nonetheless, numerous studies confirmed that the toxicity of formulated pesticides is often higher than that of the a.i.(s) themselves, confirming that the formulation agents may exert their own toxicity on non-target organisms, possibly additive or in some cases synergistic with the a.i.s [17].

Isoxaflutole (5-cyclopropylisoxazol-4-yl-2-mesyl-4-trifluoromethylphenyl-ketone), developed in 1995, is the a.i. of several commercial selective systemic herbicides for pre-emergent weed control [18]. The mode of action of isoxaflutole is the inhibition of the enzyme 4-hydroxyphenylpyruvate dioxygenase (HPPD). As a pigment inhibitor, it inhibits the biosynthesis of carotenoid pigments that protect chlorophyll from degradation by sunlight. Excessive exposure to sunlight causes photo-oxidation of chlorophyll pigments, degradation of chloroplasts, and consequent whole-plant death [19,20,21]. The molecular feature in isoxaflutole responsible for binding to HPPD has been identified to be the diketonitrile moiety formed in situ, and more potent derivatives were reported by including two of such moieties in a single molecule [22]. Nonetheless, isoxaflutole remains the sole isoxazole derivative among commercialized HPPD inhibitor herbicides, often used in herbicide-resistant weed management strategies [23,24,25]. Isoxaflutole was first registered for use on maize in 1999, and it is currently authorized in the European Union until 2034 (assessed by Sweden as rapporteur Member State) [26,27]. It was also registered in the US for use on genetically modified (GM) isoxaflutole-tolerant soybeans in 2020 [28,29,30]. With the introduction of GM crops into agricultural practice, the rate and amount of the a.i. applied increases, as shown by the worldwide use of glyphosate following the approval of tolerant GM crops [31,32]. Nonetheless, the adoption of isoxaflutole-resistant GM soy remains limited due to restrictions in the use of isoxaflutole [25].

### 1.1. Occurrence of Isoxaflutole and Its Additives in the Environment

Isoxaflutole has been detected as an emerging contaminant in surface and drinking waters due to its increasing use, together with the metabolites of diketonitrile, dichloroacetonitrile, and benzoic acid [33,34]. It has been identified as a persistent pollutant in water [21] exerting algal toxicity [35], but long-term accumulation of it and its main diketonitrile metabolite was not evidenced in semistatic water bodies [36]. Its half-life (DT_50_) water is 18 days [37] and can be substantially shorter (0.5–14 days) in soil [38] due to its leaching potential via migration through the unsaturated zone [39]. Due to the known hydrolytic decomposition of isoxaflutole and subsequent conversion to cyto- and genotoxic dichloroacetonitrile [34], the adverse biological effects of isoxaflutole can also be attributed to this latter metabolite. Due to the increasing application rates of isoxaflutole accelerated by the cultivation of isoxaflutole-resistant GM soy, its chemical load on the environment increases, yet environmental accumulation risk has not been attributed to the a.i. In cases of severe environmental pollution with pesticide a.i.s causing persistent or cumulating contamination, not only the environmental fate and toxicology of the substance, but potential means of remediation are needed to be assessed, as seen, e.g., in the case of paraquat [40] or glyphosate [32,41,42]. The environmental status of isoxaflutole is fortunately not as unfavorable, apparently manageable by certain application restrictions, and therefore the feasibility of remediation is uncalled for.

Cyprosulfamide (N-[[4-[(cyclopropylamino)carbonyl]phenyl]sulfonyl]-2-methoxybenzamide), developed in 2009, is a herbicide safener used with herbicide a.i.s including isoxaflutole. It is detected along with its metabolites in surface water, but not in ground water [43,44], and was found toxic but unlikely to cause lethality but exert adverse effects upon chronic exposure on *Daphnia magna* at relevant environmental concentrations [44]. As a common additive to frequently used pesticide formulations, cyprosulfamide and its two metabolites, its desmethyl derivative and N-cyclopropyl-4-sulfamoylbenzamide, were detected in up to 56% of the 34 surface water (but not groundwater) samples collected near cornfields in the midwestern United States. Thus, cyprosulfamide, cyprosulfamide desmethyl, and N-cyclopropyl-4-sulfamoylbenzamide were found in 25%, 19%, and 56% frequency with highest concentrations and detection rates during the growing season, and with maximum concentrations ranging between 22.0 and 5185.9 ng/L [43].

1,2-Benzisothiazoline-3-one is a preservative, disinfectant, and industrial biocide and antimicrobial agent in industrial and consumer products including formulated pesticides. Due to its widespread use, it has been detected as an aquatic environmental contaminant [45,46,47].

### 1.2. Algae as Indicators of Water Quality

Algal biomass, as an indicator of water quality and ecotoxicological impacts, can be measured using a variety of techniques. The most commonly used methods include the determination of algal cell numbers by microscope, optical density (OD), and the chlorophyll content of algal cells after alcohol extraction by spectrophotometry, and dry mass measurements [48]. The primary source of endogenous fluorescence in algae is the fluorescence signal induced by chlorophylls responsible for photosynthesis [49]. Thus, the efficiency of photosynthesis can also be characterized by fluorescence induction kinetics describing changes in the photosynthetic process and the physiological state of algal cultures [50]. The monocultures of various microalgal species (e.g., *Raphidocelis subcapitata*) are often used as test organisms in ecotoxicological studies to determine the side effects of agricultural pollutants.

This study was targeted to determine the phytotoxic effects of the herbicide a.i. isoxaflutole, its commercial formulated herbicide products (Merlin Flexx and Merlin WG75), and selected additive components used in these products (cyprosulfamide, 1,2-benzisothiazol-3(2H)-one) on *R. subcapitata* as a water quality indicator algal species, using our previously developed, modular microplate-based fluorometer prototype [35] and conventional methods. The investigation also included the assessment of the aquatic stability of the a.i. alone and within the formulation. The aim of the study was to demonstrate the effects of the formulating agents on the environmental fate and the algal toxicity of the herbicide a.i. isoxaflutole.

## 2. Materials and Methods

### 2.1. Monoculture of Raphidocelis Subcapitata

The unicellular model green algae species *R. subcapitata,* Korshikov (NIVA-CHL1) were obtained from the alga collection of the Norwegian Institute for Water Research (NIVA). This microalga is commonly applied in ecotoxicological investigations as an indicator species due to its ubiquitous distribution and high sensitivity against environmental pollution. For maintenance of the batch culture of microalgae *R. subcapitata* and the preparation of different solutions of test compounds, Zehnder 8 (Z8) [51] media were used. The culture was maintained at 23 ± 1 °C and illuminated in a 14:10 light/dark period by cool-white fluorescence tubes with a photosynthetic photon flux density of 15 µmol/m^2^/s. Fresh media were added to the cultures every two weeks.

### 2.2. Test Compounds

The effects of two isoxaflutole-based herbicide products, Merlin Flexx and Merlin WG75, were investigated. Merlin 75WG, in contrast with Merlin Flexx, is not authorized in Hungary, thus it was purchased from Slovakia. Isoxaflutole (CAS 141112-29-0), cyprosulfamide (CAS 221667-31-8), and 1,2-benzisothiazol-3(2H)-one (CAS 2634-33-5) were obtained from Merck and were of ≥97.5%, ≥98.0%, and ≥96.5%, respectively.

There appear characteristic differences between the two herbicide preparations regarding their physico-chemical properties and chemical composition. Merlin Flexx is an aqueous suspension concentrate, while Merlin WG75 is a water dispersible granulate. Although the a.i. is isoxaflutole in both preparations, the concentration of the a.i. and the chemical structure of additives applied during the formulating process are different (Table 1). In this study, the individual effects of the antidote cyprosulfamide and the antimicrobial agent 1,2-benzisothiazol-3(2H)-one; the combined effects of the a.i. and the antidote/antimicrobial agent; and the effects of the two formulated products were investigated. The combined effects of isoxaflutole + cyprosulfamide and isoxaflutole + cyprosulfamide + 1,2-benzisothiazol-3(2H)-one were investigated according to the concentration ratio in the Merlin Flexx formulated product (Table 1). As for 1,2-benzisothiazol-3(2H)-one, a concentration range (0.005–0.05%) is given by the supplier in Safety Data Sheet, and the compound was tested at 0.05% in the combination study. Information on the ingredients of the preparations is available from their safety data sheets according to the Regulation (EC) No. 1907/2006 [52,53,54].

### 2.3. Bioassay

Ecotoxicological algal growth inhibition tests were performed to investigate the possible harmful effect of the test compounds on the *R. subcapitata* monocultures according to the standardized OECD Guideline 201 [55]. The 72 h growth inhibition was described by the biomass reduction of the algal cultures, whereby optical density (OD) and chlorophyll-a (Chl-a) content were measured as endpoints. Chl-a content was detected after an extraction process that was performed according to the ISO 10260:1992 standard [56] and the Felföldy formula was applied to quantify the Chl-a content [57].

During the 72 h of tests, the following parameters were controlled: light intensity (continuous, 104.9 ± 14.9 µE/m^2^/s) by illumination (continuous, 2500 lux), temperature (22 ± 2 °C), pH of the algal Z8 medium (pH = 6–7), and intensity of stirring (continuous, 100 rpm). The ecotoxicological assays were performed in three replicates for each test substance diluted serially. In the three individual experiments, untreated control and alga suspensions exposed to different concentrations of the test substances were evaluated.

Besides the conventional parameters (OD and Chl-a content), two fluorescence-based endpoints were also measured in algal growth inhibition assays via induced fluorescence. Both the proxy of quantum efficiency of the algae photosystem PSII (F_v_*/F_p_) and the changes in the observed ratio of fluorescence decrease (Rfd*) and describe the status of the photosynthetic activity of the plants. Both parameters are calculated from fluorescence quantities observed by the FMM instrument: F_o_—non-variable fluorescence intensity; F_p_—peak fluorescence intensity, maximum fluorescence at a non-saturating light pulse; F_s_—steady-state (terminal) fluorescence. F_v_*/F_p_ and Rfd* were calculated according to the following equations: F_v_* = F_p_ − F_o_; F_d_ = F_p_ − F_s_; Rfd* = F_d_/F_s_ [35]. For measurement of the fluorescent parameters, 250 µL treated or untreated algal suspension was added into the selected wells of a 96-well microplate. Circumstances (pH, temperature, light intensity, etc.) of the ecotoxicity assay were set up according to the OECD guideline; thus, measurements of OD, Chl-a content, F_v_*/F_p_, and Rfd* were performed under the same conditions.

Growth inhibition by compounds tested in this study was investigated in two ways. Firstly, based on the equation of the dose–response curve EC_50_, values were calculated. Secondly, according to the endpoint parameters, growth inhibition rate (IR) values were determined based on the following formula: IR = (C − T)/T*100, where C and T are the density of algal cells in the control and experimental group, respectively. IR values were calculated and compared at the isoxaflutole EC_50_ equivalent concentration in all mixtures tested. Concentration of combinations were expressed by the sum of the amount of compounds in the combination.

### 2.4. Analytical Determination of Isoxaflutole Substance Loss in Its Formulated Products

The dissipation of isoxaflutole was determined under dark and light conditions in distilled water and in Z8 medium applied to maintain the algal monocultures. Dark and light conditions were applied in parallel experiments using the same treatment periods. Dissipation of isoxaflutole was determined using an analytical standard of the a.i. and samples of the two herbicide formulations Merlin Flexx and Merlin WG75. The analytical determination of isoxaflutole was performed by high-performance liquid chromatography with ultraviolet detection (HPLC-UV). A mixture of acetonitrile and water (70:30) was used as an eluent at a flow rate of 1 mL/min. Isoxaflutole was separated on a PerfectSil 100 ODS-3 column (MZ-Analysentechnik GmbH, Mainz, Germany) (150 × 4.6 mm i.d., 5 µm) at 35 °C, and UV detector signals were recorded at λ = 220 nm. The concentration of the a.i. was determined with an initial isoxaflutole-equivalent concentration of 5 µg/mL in samples collected in triplicates at 0, 9, 24, 48, 72, 96, 120, 168, 216, 264, and 336 h of treatment.

### 2.5. Instrumentation

Ecotoxicological algal growth inhibition assays on monocultures of R. subcapitata were performed in a shaking incubator (Witeg WIS-10RL, Wertheim, Germany) at controlled parameters (see Section 2.3). Chl-a content and OD values of algal suspensions were measured by a spectrophotometer (UV/VIS Camspec single beam M330, Camspec, Crawley, UK). HPLC-UV determinations were carried out using a Youngin YL9100 HPLC instrument equipped with a YL9150 autosampler (Youngin Chromass, Anyang-si, Korea).

Fluorescent parameters F_v_*/F_p_ and Rfd* were measured by the FluoroMeter Module (FMM) [35,58] equipped with an apparatus capable of holding standard-size 96-well microplates and allowing manual stepping among the wells, which is a modified version of a plant leaf fluorometer capable of measuring the excitation kinetics of Chl-a fluorescence induction besides the traditional Kautsky induction kinetics [59,60,61,62]. The main parameters of the instrument are summarized in Table 2. The limit of detection and the lower limit of quantification are 4.01 × 10^6^ and 8.12 × 10^6^ cells for *R. subcapitata*, respectively. The latter was used throughout the experiments and the kinetic curves were detected simultaneously at the two maxima of the Chl-a fluorescence (at the 690 nm red and 735 nm far-red bands) upon continuous excitation with no saturation pulses. Wavelengths applied in the determination of the OD, Chl-a content, F_v_*/F_p_, and Rfd* parameters by spectrophotometry and the induced-fluorescence method are summarized in Table 3.

### 2.6. Statistical Evaluation

Effects of tested compounds on *R. subcapitata* were described by EC_50_ ± SD mg/L values for the two formulated herbicide products (Merlin Flexx and Merlin WG75), cyprosulfamide safener, and 1,2-benzisothiazol-3(2H)-one antimicrobial agent. The results of the ecotoxicity assays were statistically analyzed by the statistical software R 4.0 (The R Foundation for Statistical Computing, Vienna, Austria). Differences between the EC_50_ values (calculated on the basis of the dose–response equation) for OD, Chl-a content, and Rfd* were detected by general linear models at a 5% significance level. Shapiro–Wilk and Levene’s or Bartlett’s test at a significance level of 5% were applied for the determination of normality and homogeneity of variance, respectively. The applicability of the fitted model was checked in each case with diagnostic plots (QQ plot, residual variances, Cook’s distance plot). Tukey’s honest significant difference (HSD) tests were performed as post hoc analyses to assess the significant differences between groups.

## 3. Results

### 3.1. Ecotoxicological Evaluation of Isoxaflutole-Based Herbicide Formulations

Ecotoxicity studies were performed to determine the effect of isoxaflutole-based formulated herbicide products (Merlin Flexx and Merlin WG75) and two additives applied in the formulating process of Merlin Flexx: cyprosulfamide safener and 1,2-benzisothiazol-3(2H)-one antimicrobial agent. The effects of these compounds were tested on *R. subcapitata* in growth inhibition assays based on the respective OECD guideline. Table 4 summarizes the highest concentration of test compounds investigated and the growth inhibition rate at these concentrations from three independent experiments. The highest concentrations were chosen to obtain a data point at the upper plateau of the sigmoid dose–response curve (concentration vs. growth inhibition). The lower concentrations investigated were sequentially diluted with a dilution factor of 1:2.

Growth inhibition was determined by measuring OD, Chl-a content, and two induced fluorescence-based parameters (F_v_*/F_p_ and Rfd*) characterizing the status of photochemical systems of the microalgal indicator species *R. subcapitata*. Concentrations resulting in 50% growth inhibition are presented in Table 5. Algal toxicity of the a.i. isoxaflutole is listed as reported in our previous study [35].

For all the test compounds and their combinations tested, significantly lower EC_50_ values (*p*: 0.005–0.030) were determined based on Chl-a content than based on the OD value. The toxicity order of the test compounds is the same for both ecotoxicological endpoints. The most toxic ingredient was found to be the a.i. isoxaflutole (EC_50(OD)_ = 0.03 ± 0.00 mg/L and EC_50(Chl-a)_ = 0.02 ± 0.00 mg/L). In the case of Merlin Flexx, the additives (see Table 1) reduced the toxicity of the a.i. by ~200-fold, as the isoxaflutole equivalent EC_50_ was found to be 6.80 ± 0.02 mg/L mg/L. The a.i. equivalent concentration is the concentration of the a.i. in the formulated herbicide product at which it exerts a 50% effect. An EC_50(OD)_ of 0.74 ± 0.20 mg/L was determined for Merlin WG75, indicating that the toxicity of isoxaflutole is substantially reduced (by one order of magnitude) by the additives present (e.g., kaolin).

For 1,2-benzisothiazol-3(2H)-one, an EC_50(OD)_ of 0.20 ± 0.00 mg/L was determined, whereas cyprosulfamide used as an antidote for isoxaflutole did not show any algal toxicity below 100 mg/L, which is the so-called limit test concentration as defined in the corresponding OECD guideline (Table 1) [55]. EC_50_ values indicated that the presence of cyprosulfamide and cyprosulfamide + 1,2-benzisothiazol-3(2H)-one resulted in ~23-fold and ~123-fold lower toxic effects of isoxaflutole, respectively, based on the OD values detected.

The determination of two photochemical parameters (F_v_*/F_p_—photochemical efficiency of the PSII photochemical system and Rfd*—fluorescence decrease ratio) was performed using the FMM fluorescence-based instrument. Both parameters characterize the functioning of the PSII photochemical system. The parameter F_v_*/F_p_ was not found to be a suitable endpoint for the study of the effect of isoxaflutole and its formulations on green microalgae, showing no concentration dependence between treatments. However, the EC_50_ values based on the Rfd* parameter were determined to be 0.02 ± 0.0 mg/L, 27.04 ± 1.41 mg/L, 0.58 ± 0.15 mg/L, >100 mg/L, and 0.18 ± 0.01 for isoxaflutole, Merlin Flexx, Merlin WG75, cyprosulfamide and 1,2-benzisothiazol-3(2H)-one, respectively. The results were significantly lower (*p*: 0.009–0.040) for all the compounds and their mixtures tested compared to EC_50_ values determined based on OD and Chl-a content (the difference was not significant in the case of Merlin Flexx and isoxaflutole compared to Chl-a content), suggesting that the vitality index determined by induced fluorescence is a more sensitive parameter compared to the determination of OD and Chl-a content after alcohol extraction. It also provides additional information on the effect of the test substance. Growth inhibition rates calculated for isoxaflutole EC_50_ values equivalent for herbicide products and combinations of the a.i. and additives are presented in Table 6.

### 3.2. Analytical Determination of Substance Loss of Isoxaflutole

**Figure 1:**
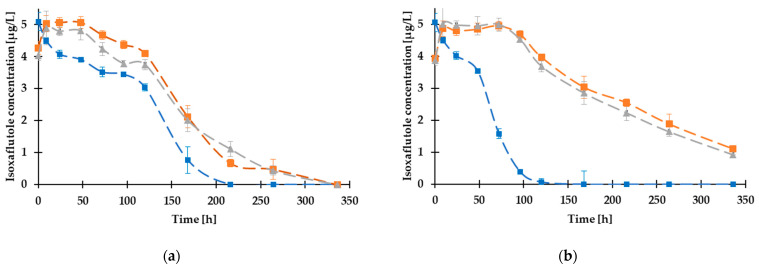
Dissipation of isoxaflutole in the form of an analytical standard (blue lines) and formulated herbicide products Merlin Flexx (grey lines) and Merlin WG75 (orange lines) under (**a**) light and (**b**) dark conditions. Data for isoxaflutole in form of an analytical standard under light conditions were published earlier [35].

Isoxaflutole, as the replacement compound for atrazine, has been detected as an emerging water pollutant in the US and the EU [33,63]. For environmental risk assessment, it is crucial to obtain information regarding the fate of pollutants in different environmental matrices. The aim of this analytical determination was to ensure the accuracy of algal toxicity tests, the stability of the compound during the test, and to gather information about the dissipation time (DT_50_) value of isoxaflutole in the presence of additives applied in the herbicide formulations. The dissipation of isoxaflutole in the form of an analytical standard and also in its formulated products was determined in distilled water and Z8 medium, under dark and light conditions with initial isoxaflutole and isoxaflutole-equivalent concentrations of 5 µg/L. The results showed no apparent dissipation of isoxaflutole in distilled water within 2 weeks. For Z8 media, the results are presented in Figure 1. In the two formulations, the analytical determination of the samples has not immediately reached the initial concentration of 5 µg/L at 0 h due to a slight time delay of micelle formation of isoxaflutole with the surfactant additives in the formulation. Considerable differences appeared regarding the dissipation of isoxaflutole in pure form and formulated herbicides both under dark and light conditions. Dissipation of the a.i. isoxaflutole as an analytical standard was completed on the 216th hour (DT_50_ = 131 h) and the 120th (DT_50_ = 59 h) under light and dark conditions, respectively. In both conditions, the additives in the formulations Merlin Flexx and Merlin WG75 (Table 1) contributed to a longer dissipation process. Under light conditions, the substance loss was completed on the 336th hour for both formulations, with DT_50_ values of 154 h and 158 h for Merlin Flexx and Merlin WG75, respectively. In contrast, under dark conditions, the dissipation remained incomplete during the experiment. DT_50_ values were found to be 194 h and 222 h for Merlin Flexx and Merlin WG75, respectively.

The additives in formulated herbicides had a considerable influence on the dissipation of isoxaflutole, as they delayed the dissipation time and resulted in higher DT_50_ values. However, results indicated that the differences between dark and light circumstances were more remarkable than the chemical characteristics of the additives in the two formulated products.

## 4. Discussion

The mode of action of isoxaflutole is the inhibition of the enzyme 4-hydroxyphenylpyruvate dioxygenase, thus the quite high toxicity of the a.i. to the algal species *R. subcapitata* is not surprising, as it acts on all green plant organisms. Isoxaflutole has been shown to exert phytotoxicity on green algae: its 72 h EC_50_ values have been reported to range between 0.003 and 0.380 mg/L on freshwater green or diatom algae and duckweed [64], 0.030–1.71 mg/L on *R. subcapitata* [18,35,65] with 0.12 mg/L on 120 h exposure time [66], and 0.016–0.140 mg/L on *Selenastrum capricornutum* [18,64]. Similar inhibitory effects of isoxaflutole have been demonstrated in a plant spectral analysis system for herbicide efficacy using a terrestrial model weed crabgrass (*Digitaria ciliaris*) as a test plant and chlorophyll fluorescence as an endpoint [67]. The additional 96-well microplate-based biotest detected the effects on chlorophyll fluorescence, but instead of applying fluorimetry as in our case, it used a semiconductor device, a complementary metal oxide semiconductor camera. However, wide variability is seen among aquatic plant organisms regarding their sensitivity to this a.i. [68]. In our present study, a reduced toxicity of the two formulated preparations compared to the a.i. was observed, due to the combined effect of the a.i. and the additives.

Although it has already been reported for various pesticide a.i.s that the presence of additives in pesticide formulations can alter the toxicity of the a.i. [69,70,71,72,73], our results are the first that demonstrate the same phenomenon for isoxalfutole-based formulations. Moreover, the individual toxicity of numerous additives has also been published [74,75,76], among which the first generation of polyethoxylated amines has been the focus recently. As a result of numerous independent studies [74,77,78,79], this surfactant-type was banned in 2016 as a co-formulant in the European Union and was progressively replaced by other surfactants, ethoxylated etheramines, which exhibited lower toxicity on non-target organisms [80,81]. For both, the composition of the preparations and the physico-chemical characteristics of the additives were different, which exerted significantly different toxic effects. Thus, the influence of additives applied in formulated products on the toxic properties of the herbicide formulations is evident.

Nonetheless, the views on the health safety of isoxaflutole have not been unambiguously positive. As early as during its initial assessment and subsequent toxicological evaluation, the US EPA classified the carcinogenic potential of isoxaflutole in 1997 as “likely” to be carcinogenic to humans by all routes of exposure [82], and stated later in 2011: “In carcinogenicity studies, isoxaflutole induced liver and thyroid tumors in rats and liver tumors in mice. Isoxaflutole was classified as ‘‘likely to be a human carcinogen’’. The method of quantification was linear cancer slope factor (Q1*).” [83]. The European Food Safety Authority (EFSA) proposed to classify it as a carcinogen category 2 (suspected human carcinogen) due to observed thyroid follicular cell adenoma in rats and liver carcinoma in mice [65]. These tumor-inducing effects of isoxaflutole are suggested to lay on the grounds of endocrine disruption [84]. Thyroid tumor induction was considered to occur via a possible disruption of the thyroid–pituitary hormonal feedback mechanisms [82], and since this proposed mode of thyroid tumor induction can apply to humans as well, a warning has recently been published that this mode of action of isoxaflutole is of public health relevance [85]. However, approval of the a.i. was approved as no experimental evidence proved the assumption of the carcinogenicity of isoxaflutole on humans.

Information on the effects of both formulations regarding the toxicity to aquatic plant is only available in their MSDS [52,53]. For Merlin Flex and Merlin WG75, EC_50_ values are described for *R. subcapitata* and *Desmodesmus subspicatus* green algal species, respectively. Varying by the endpoints detected in this study, a 4.1–5.1-fold lower toxicity for Merlin Flexx, and a minimum 46.9–59.8-fold higher toxicity for Merlin WG75 on *R. subcapitata* were found compared to data in the MSDSs. The difference in the case of Merlin WG75 is due to the different sensitivities of different algal species.

The antimicrobial additive 1,2-benzisothiazol-3(2H)-one in Merlin Flexx is highly toxic to aquatic ecosystems and its presence in surface waters has been demonstrated [45,46,47]. Wang et al. studied the effect of the additive on three species of green algae. For *Scenedesmus* sp. LX1, *Chlorella* sp. HQ, and *Chlamydomonas reinhardtii,* the detected EC_50_ values were 1.70, 0.41, and 1.16 mg/L, respectively, indicating the differences in sensitivity between species and the importance of including more species in the environmental risk assessment. It was found that the primary effect of 1,2-benzisothiazol-3(2H)-one was to inhibit photosynthesis. This inhibitory effect appeared reversible at exposure in the concentration range of 1–30 mg/L [86]. In our study, *R. subcapitata* appeared to be 2–8-fold more sensitive to the additive (EC_50_ = 0.20 ± 0.00 mg/L) than the above-mentioned algal species. The cyprosulfamide additive did not show any toxic effect at a concentration of 100 mg/L on the algae species tested. As an antidote, it protects the plant against the toxic effect of the a.i. isoxaflutole, an effect which was presumably also observed in *R. subcapitata*. Its lower toxicity compared to Merlin Flexx is probably due to this mechanism of action. A similar moderating effect of another safener benoxacor on the toxicity of the herbicide a.i. iodosulfuron–methyl–sodium was reported on *Desmodesmus subspicatus* [87].

Our dissipation study of isoxaflutole in the forms of analytical standard and formulated products indicated that the presence of additives in the formulation prolonged the DT_50_ value of isoxaflutole in water. In contrast to the ecotoxicity experiment on *R. subcapitata* alga species, in the dissipation study, the composition of the formulated products and the physico-chemical characteristics of the additives did not significantly influence the dissipation time. The alteration of dissipation time when the a.i. and the additive are present in water samples at the same time has been also published for neonicotinoid-type and glyphosate a.i.s. in surface water [77,88,89] and for esfenvalerate in seawater samples [90], and slight differences in the kinetics of dissipation were observed for the herbicide a.i. sulcotrione alone and in the formulation Tangenta^®^ [91]. For the determination of isoxaflutole at low concentrations, liquid chromatography coupled with tandem mass spectrometry (LC-MS/MS) is the analytical method of choice with LOD and LOQ values low enough to detect the a.i. under the maximum residue level in food and feed products or the maximum individual limit (0.1 μg/L) in surface water [92,93,94,95]. In our study, a simpler LC-UV method was applied for the detection of isoxaflutole dissipation, as our aim was only to determine its DT_50_ values, not the a.i. at very low concentrations. In an environmental and ecotoxicological impact assessment study of various herbicide mixtures of 20 a.i.s, carried out by an Italian researchers, isoxaflutole itself and combinations containing it were found to be most favorable regarding the pesticide index (a specific score based on selected pesticide indicators including water affinity, soil mobility, soil persistence, water persistence, percolation index, partition coefficient, and volatility), while among the worst ones regarding the priority index for surface and ground waters (a specific score based on sales data, type of application, environmental distribution, and soil persistence) [96]. A recent survey reported the use of isoxaflutole to be 193 tons/year in the US only in 2018, being the third-most highly used HPPD inhibitor in maize with a single application annually at an application dosage of 72 g/ha [29]. Lipophilicity of the two additives cyprosulfamide and 1,2-benzisothiazol-3(2H)-one are similar (logK_ow_ 0.55 [44] and 0.70 [97], respectively); therefore, their potential effects on the solubility features of the two orders of magnitude and more lipophilic feature of isoxaflutole (logK_ow_ 2.34 [18]) are similar, and the concentration of 1,2-benzisothiazol-3(2H)-one is 400–4000-fold lower than that of isoxaflutole.

## 5. Conclusions

The ecotoxicological effect of two isoxaflutole-based formulations (Merlin Flexx and Merlin WG75) and two additives (cyprosulfamide safener and 1,2-benzisothiazol-3(2H)-one antimicrobial agent) were determined on the growth inhibition of *R. subcapitata* green algal indicator species. The combined effects of the a.i. isoxaflutole with cyprosulfamide and cyprosulfamide + 1,2-benzisothiazol-3(2H)-one were also determined to obtain a better insight to the effect of herbicide formulations and their ingredients. After the ban of atrazine (in 2004 in the EU), isoxaflutole has become its replacement a.i.; thus, the application of isoxaflutole-based formulations is increasing. As a consequence, the environmental burden from the a.i. and additives applied in its formulations is on the rise as well. For risk assessments, it is essential to know the fate of the ingredients in different environmental matrices and the dissipation characteristics. Our results indicated that the additives in Merlin Flexx and Merlin WG75 influence the DT_50_ values in aqueous media and the toxicity of the isoxaflutole-based formulations. The toxicity of isoxaflutole was lower in the presence of the tested safener and antimicrobial agent presented in Merlin Flexx. Moreover, the presence of other additives in the formulation resulted in an even lower toxicity of the formulation. In contrast, the additives in the formulation prolonged the dissipation time of the a.i.; thus, the environmental load represented by the ingredients is longer if they are present together in the same media.

Results of ecotoxicity assays showed that the most sensitive endpoint was the induced fluorescent-based parameter Rfd* that characterizes the aptness of the photochemical system of the microalgae tested well. The benefit of the application of Rfd* values is that the determination requires no sample preparation compared to the measurement of Chl-a content. The least sensitive parameter was the optical density. However, the toxicity order of the tested compounds was the same for all the three endpoints. In this study, the corresponding OECD Guideline was applied for the detection of the ecotoxicological effects of additives in isoxaflutole-based herbicide formulations. Thus, the results are limited to the growth inhibition of test compounds on *R. subcapitata* determined by conventionally applied endpoints (OD and Chl-a content). In addition, a fluorescence-based parameter (Rfd*) was also assessed to obtain information regarding the possibly detrimental effects of the compounds and their combinations tested on the PSII photochemical system. For a more complex risk assessment study, investigation of the test compounds in field studies are required and biochemical and physiological experiments can contribute for a better understanding of the mode of action of test compounds.

## Figures and Tables

**Table 1:** Composition of the isoxaflutole-based herbicide products.

Herbicide Products	Concentration (%)	CAS No ^1^	Function in the Herbicide Product
Merlin Flexx			
isoxaflutole	20.3	141112-29-0	active ingredient
cyprosulfamide	20.3	221667-31-8	additive: antidote
1,2-benzisothiazol-3(2H)-one	0.005–0.05	2634-33-5	additive: antimicrobial
D-glucopyranose, oligomer, C9-11-alkyl glycosides	3–10	132778-08-6	additive: surfactant
glycerin	>1	56-81-5	humectant, antigelling, and antifreeze agent
Merlin WG75			
isoxaflutole	75	141112-29-0	active ingredient
sodium diisopropylnaphthalene sulphonate	3–10	1322-93-6	additive: surfactant
lignosulfonic acid, sodium salt, sulfomethylated	3–10	68512-34-5	suspension/emulsion stabilizer
kaolin	>1	1332-58-7	carrier
pyrogenic (fumed) amorphous silica	>1	112945-52-5	carrier

^1^ Chemical Abstract Service Number.

**Table 2:** Features of the FluoroMeter Module applied in ecotoxicity assays on *Raphidocelis subcapitata*.

FluoroMeter Module	Feature/Type	Role
actinic light source	635 nm laser diode with 256-step digital optical power adjustment	excitation
interference filters	NT43-089 for 690 nm and NT43-091 for 730 nm, full width at half maximum of 10 nm each ^1^	separation of the detection wavelengths and eliminate the scattered illumination light
cut-off filters	RG665 for 665 nm ^1^	
photodetector	Low-noise PIN photodetectors (SD-200-14-21-241) ^2^	photocurrent-to-voltage conversion
electrometer amplifier	OPA129 ^3^	
signal amplifier	AD620 ^4^	
digitalizer	12-bit ADC (AD7864-2) ^4^	signal digitalization
computer	single-board computer (CMD16686GX) ^5^	firmware, storage of data

^1^ Edmund Optics, Barrington, NJ, USA; ^2^ Laser Components, Olching, Germany; ^3^ Texas Instruments, Dallas, TX, USA; ^4^ Analog Devices, Willington, MA, USA; ^5^ Real Time Devices, Wickwar, UK.

**Table 3:** Wavelengths applied in determination of endpoint parameters of ecotoxicological algal growth inhibition assays.

Method	Parameter Measured	Wavelength ^1^
spectrophotometry	Optical density	detection: 750 nm
spectrophotometry	Chl-a content	detection: 750 nm ^2^, 666 nm ^3^, 653 nm ^4^
induced-fluorescence	F_v_*/F_p_	excitation: 635 nm detection: 690 nm, 735 nm
induced-fluorescence	Rfd*	excitation: 635 nm detection: 690 nm, 735 nm

^1^ Applied in the measurement procedure of the given parameter. ^2^ Degree of turbidity. ^3^ First detection wavelength of Chl-a. ^4^ Second detection wavelength of Chl-a.

**Table 4:** Growth inhibition rates at the highest concentrations investigated in this study.

Substance	The Highest Concentration Investigated in This Study (mg/L)	IR (%)
active ingredient		
isoxaflutole	0.064	98.0 ± 1.0
herbicide products		
Merlin Flexx	80	96.3 ± 0.6
Merlin WG75	1.3	96.0 ± 4.7
combinations		
cyprosulfamide	100	21.3 ± 1.5
1,2-benzisothiazol-3(2H)-one	0.6	
isoxaflutole + cyprosulfamide	10 + 10	96.3 ± 1.5
isoxaflutole + cyprosulfamide + 1,2-benzisothiazol-3(2H)-one	5 + 5 + 0.025	95.7 ± 3.0

**Table 5:** Ecotoxicological effects of the active ingredient isoxaflutole and its formulations on the growth of the algal species *Raphidocelis subcapitata* as determined by optical density (OD), Chl-a content, and Rfd*.

Substance	Method of Detection ^1^	EC_50_ ± SD (mg/L)	Isoxaflutole-Equivalent EC_50_ ± SD (mg/L) ^2^
active ingredient			
isoxaflutole ^3^	OD	0.03 ± 0.00	0.03 ± 0.00
	Chl-a	0.02 ± 0.00	0.02 ± 0.00
	Rfd*	0.02 ± 0.00	0.02 ± 0.00
herbicide products			
Merlin Flexx	OD	33.3 ± 1.10	6.80 ± 0.20
	Chl-a	28.52 ± 0.32	5.84 ± 0.32
	Rfd*	27.04 ± 1.41	5.48 ± 0.48
Merlin WG75	OD	0.74 ± 0.20	0.55 ± 0.17
	Chl-a	0.64 ± 0.10	0.50 ± 0.07
	Rfd*	0.58 ± 0.15	0.43 ± 0.11
additives			
cyprosulfamide	OD	>100	–
	Chl-a	>100	–
	Rfd*	>100	–
1,2-benzisothiazol-3(2H)-one	OD	0.20 ± 0.00	–
	Chl-a	0.10 ± 0.01	–
	Rfd*	0.18 ± 0.01	–
combinations			
isoxaflutole + cyprosulfamide	OD	1.37 ± 0.04	0.69 ± 0.02
	Chl-a	1.22 ± 0.06	0.61 ± 0.03
	Rfd*	0.91 ± 0.07	0.46 ± 0.04
isoxaflutole + cyprosulfamide + 1,2-benzisothiazol-3(2H)-one	OD	7.43 ± 0.58	3.71 ± 0.29
	Chl-a	6.33 ± 0.44	3.12 ± 0.22
	Rfd*	5.64 ± 0.45	2.81 ± 0.22

^1^ Methods of detection of algal biomass growth of *R. subcapitata* determined by optical density (OD), by extracted Chl-a content, and by the observed ratio of fluorescence decrease of the algal photosystem PSII (Rfd*) detected by our modular microplate-based fluorometer prototype [35]. ^2^ The 72 h EC_50_ value for the active ingredient (a.i.) represents the concentration of the a.i. present in the mixture or product that exerts a 50% effect in the given biotest. ^3^ Published earlier by Lázár et al., 2023 [35].

**Table 6:** Growth inhibition rate of the herbicide products and combinations at isoxaflutole EC_50_ equivalent concentrations.

Substance	Isoxaflutole EC_50_ Equivalent Concentration (mg/L)	IR (%)	
active ingredient			
isoxaflutole	0.03	OD	50.0
	0.02	Chl-a	50.0
	0.02	Rfd*	50.0
herbicide products			
Merlin Flexx	0.15	OD	0.22
	0.1	Chl-a	0.17
	0.1	Rfd*	0.18
Merlin WG75	0.04	OD	3.64
	0.03	Chl-a	3.00
	0.03	Rfd*	3.49
combinations			
isoxaflutole + cyprosulfamide	0.06	OD	2.17
	0.04	Chl-a	1.64
	0.04	Rfd*	2.17
isoxaflutole + cyprosulfamide + 1,2-benzisothiazol-3(2H)-one	0.065	OD	0.88
	0.045	Chl-a	0.72
	0.045	Rfd*	0.8

## Data Availability

The data presented in this study are available on request from the corresponding author. The data are not publicly available due to privacy reasons.
